# Simulated-Physiological Loading Conditions Preserve Biological and Mechanical Properties of Caprine Lumbar Intervertebral Discs in *Ex Vivo* Culture

**DOI:** 10.1371/journal.pone.0033147

**Published:** 2012-03-13

**Authors:** Cornelis P. L. Paul, Hendrik A. Zuiderbaan, Behrouz Zandieh Doulabi, Albert J. van der Veen, Peter M. van de Ven, Theo H. Smit, Marco N. Helder, Barend J. van Royen, Margriet G. Mullender

**Affiliations:** 1 Department of Orthopedic Surgery, VU University Medical Center, Amsterdam, The Netherlands; 2 Research institute MOVE, VU University Medical Center, Amsterdam, The Netherlands; 3 Faculty of Oral Cell Biology, Academic Centre of Dentistry Amsterdam, Amsterdam, The Netherlands; 4 Department of Physics and Medical Technology, VU University Medical Center, Amsterdam, The Netherlands; 5 Department of Epidemiology and Biostatistics, VU University Medical Center, Amsterdam, The Netherlands; 6 Skeletal Tissue Engineering Group Amsterdam, VU University Medical Center, Amsterdam, The Netherlands; 7 Department of Plastic, Reconstructive and Hand Surgery, VU University Medical Center, Amsterdam, The Netherlands; University of Western Ontario, Canada

## Abstract

Low-back pain (LBP) is a common medical complaint and associated with high societal costs. Degeneration of the intervertebral disc (IVD) is assumed to be an important causal factor of LBP. IVDs are continuously mechanically loaded and both positive and negative effects have been attributed to different loading conditions.

In order to study mechanical loading effects, degeneration-associated processes and/or potential regenerative therapies in IVDs, it is imperative to maintain the IVDs' structural integrity. While in vivo models provide comprehensive insight in IVD biology, an accompanying organ culture model can focus on a single factor, such as loading and may serve as a prescreening model to reduce life animal testing. In the current study we examined the feasibility of organ culture of caprine lumbar discs, with the hypothesis that a simulated-physiological load will optimally preserve IVD properties.

Lumbar caprine IVDs (n = 175) were cultured in a bioreactor up to 21 days either without load, low dynamic load (LDL), or with simulated-physiological load (SPL). IVD stiffness was calculated from measurements of IVD loading and displacement. IVD nucleus, inner- and outer annulus were assessed for cell viability, cell density and gene expression. The extracellular matrix (ECM) was analyzed for water, glycosaminoglycan and total collagen content.

IVD biomechanical properties did not change significantly with loading conditions. With SPL, cell viability, cell density and gene expression were preserved up to 21 days. Both unloaded and LDL resulted in decreased cell viability, cell density and significant changes in gene expression, yet no differences in ECM content were observed in any group.

In conclusion, simulated-physiological loading preserved the native properties of caprine IVDs during a 21-day culture period. The characterization of caprine IVD response to culture in the LDCS under SPL conditions paves the way for controlled analysis of degeneration- and regeneration-associated processes in the future.

## Introduction

Low-back pain (LBP) is the most common medical complaint in Western society, possibly leading to an incapacitating condition and encompassing considerable ensuing socio-economic costs [1]. It is widely recognized that multiple factors underlie the complex pathology of LBP. Intervertebral disc (IVD) degeneration, or degenerative disc disease (DDD), has been associated with LBP [2]–[5] and recent large population based studies provide strong evidence for their correlation [6]. Presently, the only options for patients with symptomatic disc degeneration are conservative treatments, such as physical therapy [7], pain medication [8] and acupuncture [9], or surgical salvage procedures involving removal of the disc followed by fusion or arthroplasty [10], [11].

Various new treatment strategies are being developed to halt the progression of degeneration or even to regenerate the intervertebral disc. This is challenging as DDD itself is considered a multi factorial process [12]. Many risk factors have been identified such as trauma to the spine [13], [14], aging [15]–[17], loss of nutrient supply to the disc [18], and genetic predispositions [19]–[24]. Mechanical loading of the intervertebral disc is considered to be a major extrinsic cause of intervertebral disc degeneration [25]–[28]. Yet, load bearing is the primary function of the IVD, with discs continuously being under considerable pressure even during rest. Moreover, mechanical loading is a natural stimulus to chondrocytes and regarded to be essential for maintenance of the cartilaginous matrix [25],[29]–[34].

In order to develop therapies against DDD more detailed knowledge is needed on the influence of loading on the preservation, degeneration and regeneration of the IVD [4]. This cannot be adequately investigated in cell culture models, because these cannot mimic the specific tissue composition and exceptional physical conditions of the IVD. In vivo animal models such as described in earlier studies from our group, lack close control and monitoring of mechanical conditions of the IVD. Several organ culture models with IVDs of various animal species have been introduced to study disc function and the role of different etiological factors involved in DDD [35]–[39]. These models vary in their relevance to the human situation with regard to IVD dimensions, biomechanical properties, and cellular and matrix composition [40].

Ideally, an ex vivo model would implement a large species lumbar IVD comparative in biological and mechanical properties to the human IVD [41], as a precursory platform to an *in vivo* DDD model for follow-up studies. As we have shown in recent publications, the goat IVD closely resembles the human IVD with respect to mechanical properties [42]. Moreover, as in human IVDs, the caprine IVD lacks notochordal cells, which also makes the goat IVD comparable from a biological perspective. Together with our well established goat *in vivo* IVD degeneration and herniation model, the caprine lumbar IVD is an excellent candidate for *ex vivo* studies on disc degeneration and regeneration [43]–[46].

**Figure 1:**
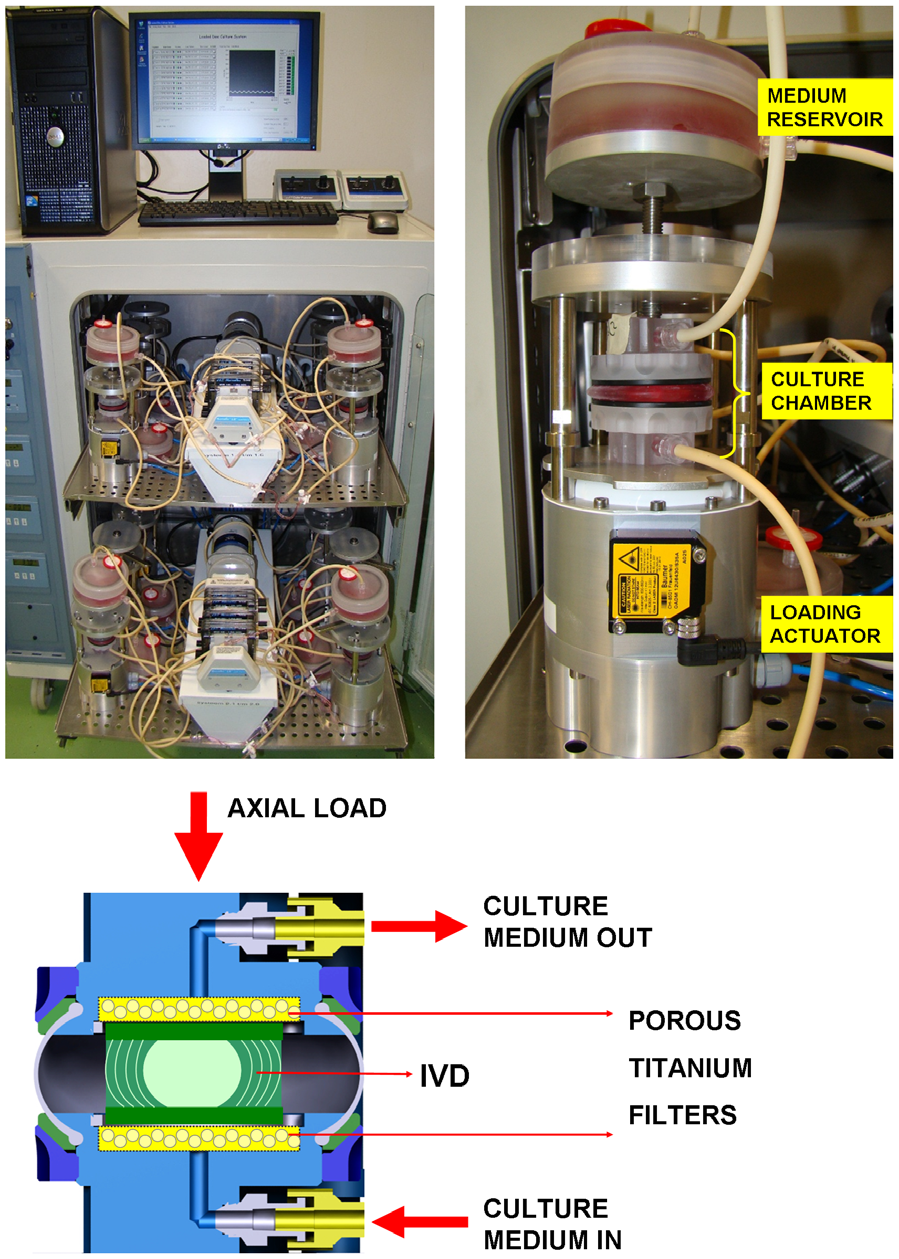
The Loaded Disc Culture System. The Loaded Disc Culture System (LDCS; upper left), a single actuator (upper right) with culture chamber and reservoir, and a schematic cross-section (bottom) of a culture chamber with IVD.

Therefore we have developed a bioreactor, the Loaded Disc Culture System (LDCS; figure 1), designed to culture entire large IVDs (i.e. IVD with cartilaginous endplates), conserving IVD cells in their native environment. The LDCS allows for precise control and monitoring of oxygen- and nutrient supply, as well as mechanical loading conditions via a force-feedback loop. Providing a platform to study the interaction between disc loading and IVD biology. Moreover, an ex vivo model may serve as a prescreening platform of future therapeutics prior to in vivo testing on life animal.

The objective of the current study is to test the feasibility of ex vivo culture of caprine lumbar IVDs in the LDCS over a 21 day period. More specifically, we aim to characterize the IVDs response to culturing with and without loading. We hypothesize that applying simulated-physiological loading will be appropriate to maintain the goat IVD properties.

## Results

### IVD Geometry and Biomechanics

The average calculated cross-sectional area of the mid-lumbar IVD levels L2–L3, L3–L4 and L4–L5 was 4.0±0.65 cm^2^ and did not differ significantly between levels. For homogeneity of loading pressure, these IVDs were assigned to the loaded culture conditions. IVDs at levels L1–L2 and L5–L6 showed larger deviations in calculated cross-sectional area (respectively 3.76±0.64 cm^2^ and 4.11±0.54 cm^2^), therefore these IVDs were used as day 0 controls or unloaded culture controls.

**Figure 2:**
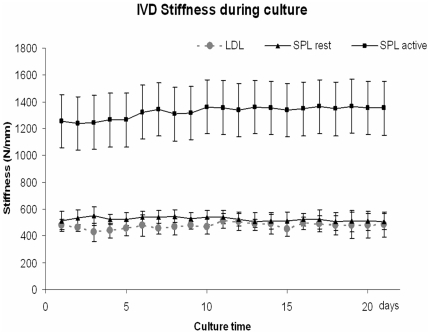
IVD stiffness during culture. Mean IVD stiffness (N/mm) ±SD as calculated from disc loading and displacement measurements during the culture period in the LDCS. The gray dotted line represents IVD stiffness from the discs cultured under LDL condition. The black lines represent the SPL loaded IVD stiffness, respectively during the rest phase (triangles) and active phase (squares) of the diurnal SPL regime.

In the unloaded and LDL groups the overall disc height did not change after the first culture day, whereas in the SPL group disc height decreased with 0.15±0.05 mm over 21 days. IVD stiffness was calculated in the dynamically loaded groups (i.e. LDL and SPL). Initial IVD stiffness varied considerably between individual goats and between levels. The stiffness magnitudes increased when higher magnitude loads were applied, but stiffness did not change significantly over time. In the LDL group overall average stiffness during culture was 474.5±58.5 N/mm and in the SPL group during the rest and active phase, stiffness was respectively 524.6±57.7 N/mm and 1323.5±251 N/mm. Figure 2 shows the average disc stiffness during culture under LDL and SPL in the LDCS over a 20 day period.

### Histology

**Figure 3:**
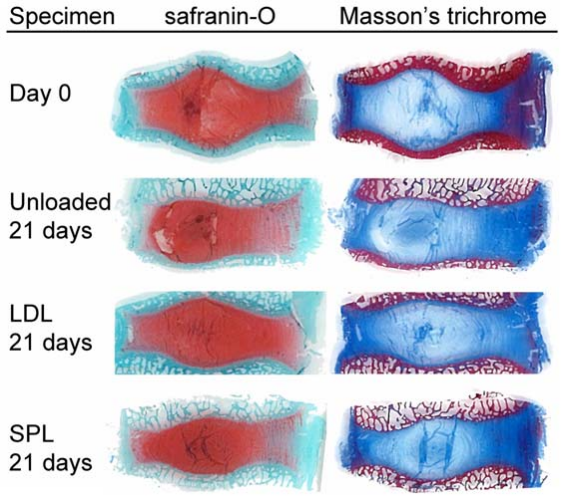
Histology of IVD specimens. Midsagittal paraffin sections of IVD specimens (anterior side of IVDs facing right) at day 0 and after 21 days of culture at unloaded, LDL and SPL loading conditions. The left column shows safranin-O stained sections for the proteoglycan distribution (red). In the right column Masson's trichrome staining for distribution of the collagen (blue) is shown.

Figure 3 shows representative midsagittal IVD sections stained with safranin-O (left column) and Masson's trichrome (right column). Sections illustrate the gross morphology of the IVD specimens, showing the nucleus, inner- and outer annulus region from the center to the periphery of the disc. On the safranin-O sections proteoglycans are stained red and on the Masson's trichrome sections collagen is stained blue. No clear differences in structure or staining intensity of matrix components in the nucleus or annulus could be noted when comparing the day 21 sections of the experimental groups and baseline controls (day 0).

### Cell viability and density

**Figure 4:**
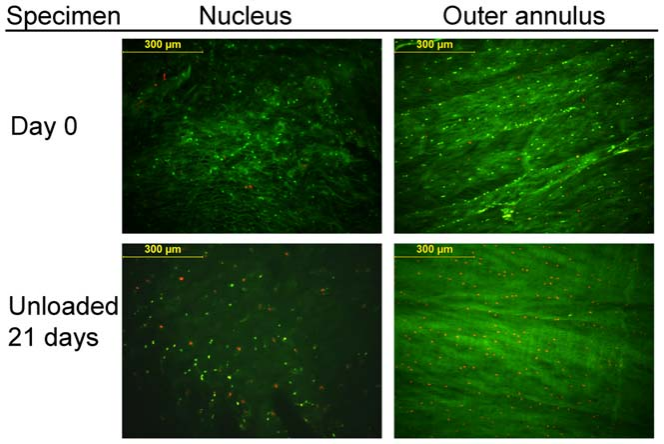
Fluorescent live/dead staining. Representative images at 10× magnification of the nucleus and outer annulus regions of IVD. Shown is fluorescent staining on transverse cryosections of live cells with (green) and dead cells (red). The upper two images are taken from a fresh disc, stained directly after dissection from the lumbar goat spine at baseline control (day 0). The lower two images are taken from an IVD after 21 days of culture in the LDCS under unloaded conditions.

**Figure 5:**
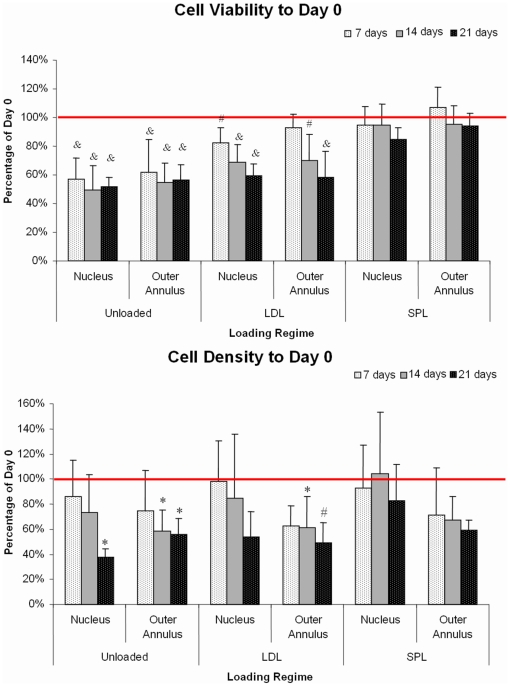
Quantitative cell biology. Cell viability (above; live cells/total cells +SD) and cell density (below; mean total cells/mm^2^ +SD) as percentage of day 0 for each loading condition in the nucleus and outer annulus region after 7, 14 and 21 days of culture in the LDCS. *P* values comparing experimental groups with day 0 group in a linear mixed model with Bonferroni *post-hoc* testing: *p<0.05; ^#^ *p*<0.01; ^&^ *p*<0.001.

Figure 4 shows representative images of CTG-PI stained NP and oAF IVD cryosections. Cell viability in fresh discs did not differ significantly between disc levels or regions. Overall mean viability (±SD) at day 0 in the nucleus was 79.0% (±10.7%) and 75.1% (±11.5%) in the outer annulus. Table 1 shows cell viability and cell density measures for all experimental groups and time-points for nucleus and outer annulus region. Values for the inner annulus were in between those found for the nucleus and outer annulus region (data not shown). The linear mixed model showed that cell viability and cell density depend significantly on the loading regime, the culture period, and their interaction. A significant effect of culture duration on cell viability was found in all disc regions for unloaded and LDL regime, but not for SPL. Both cell viability (i.e. percentage of live cells) and cell density (i.e. the total number of cells per area) decreased significantly in the unloaded and LDL groups relative to day 0 in all regions of the IVD. In the unloaded group a steep loss of live cells was already apparent within the first week of culture, after which viability seemed to stabilize. However, the cell density reveals an ongoing decrease in total cells at 14 and 21 days in both nucleus and outer annulus. In the LDL group the loss in cell viability and density was more gradual, whereas both parameters were maintained in the simulated-physiological load group when compared to day 0 (figure 5).

**Table 1:** Cell Viability and Cell Density.

Group		Cell Viability (%)				Cell Density (*cells/mm^2^*)			
		Nucleus		Outer Annulus		Nucleus		Outer Annulus	
Control	day 0	79.0	±10.7	75.1	±11.5	193.5	±82.5	324.2	±121.5
Unloaded	7 days	44.9	±11.7^&^	46.4	±17.2^&^	166.5	±55.9	241.8	±104.2
	14 days	39.1	±13.3^&^	41.0	±10.1^&^	141.9	±58.8	189.3	±54.6*
	21 days	40.7	±5.4^&^	42.2	±8.4^&^	72.5	±13.6*	179.8	±42.0*
LDL	7 days	65.1	±8.4^#^	69.9	±6.9	190.1	±62.9	202.4	±53.4*
	14 days	54.2	±9.9^&^	52.7	±13.7^#^	164.1	±98.6	198.8	±81.0*
	21 days	46.8	±6.7^&^	43.7	±13.5^&^	104.3	±38.9	157.9	±53.9^#^
SPL	7 days	74.6	±10.4	80.2	±10.9	179.8	±66.0	242.5	±121.1
	14 days	74.7	±11.9	71.4	±9.8	201.1	±96.0	244.3	±61.1
	21 days	66.8	±6.7	70.7	±6.7	160.6	±55.4	238.3	±27.1

Table 2 Mean cell viability (percentage live cells ±SD) and mean cell density (cells/mm2 ±SD) per experimental group, region and test duration. *P* values comparing experimental groups with day 0 group in a linear mixed model with Bonferroni post-hoc testing: *p<0.05; ^#^ *p*<0.01; ^&^ *p*<0.001.

### Gene Expression

For each gene of interest, expression levels at day 0 and after 7, 14 and 21 days of culture were compared for the three loading conditions (unloaded, LDL and SPL). Statistical analyses showed expression of all genes was highly dependent on the type of load on the IVDs. Within each load group, there was no effect observed for culture duration, i.e. expression of each gene of interest at day 7, 14 and 21 days did not differ significantly within a loading regime. For this reason, it was decided to compare the mean expression levels of each gene at day 0 and the average mean expression levels for this gene per load regime during culture. For these analyses time was dichotomized; the first category corresponded to the day 0 measurements and the second category to measurements made on day 7, 14 and 21.

**Figure 6:**
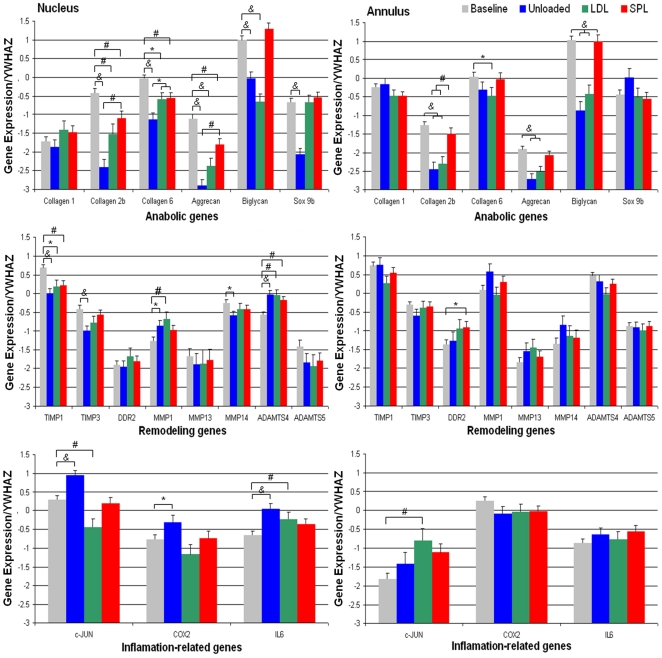
Gene expression. Relative gene expression (log means ± SEM, normalized to YWHAZ) in the baseline (day 0; grey), unloaded (blue), LDL (green) and SPL (red) group. Shown in the left column is the data for the nucleus region and in the right column for the outer annulus region. Graph rows from top to bottom show respectively the anabolic, remodeling and inflammation-related genes. Brackets indicate significant statistical differences between groups when comparing in a linear fixed model with Bonferroni post-hoc testing. *P* values are indicated by: ^*^ *p*<0.05; ^#^ *p*<0.01; ^&^ *p*<0.001.

For the anabolic genes all culture groups showed significant down-regulation of collagen type 2, collagen type 6 and aggrecan gene expression in the nucleus, when compared to day 0. The unloaded and LDL group also showed down-regulation of these genes in the outer annulus region, together with down-regulation of biglycan. Interestingly, only the unloaded group displayed a significant down-regulation of Sox9 in the nucleus. Also, when comparing the anabolic gene expressions of the unloaded and LDL group to the SPL group, we found that collagen 2b, aggrecan and biglycan are significantly down-regulated in both nucleus and outer annulus (figure 6).

For the remodeling genes in the unloaded and LDL group there was significant down-regulation of TIMP1, together with up-regulation of MMP1 and ADAMTS4 in the nucleus region. Also, in the SPL group TIMP1 was down-regulated and ADAMTS4 up-regulated, but significantly less when compared to the unloaded or LDL group. The inflammation-related genes c-JUN, COX2 and IL6 showed significant up-regulation in the nucleus region of the unloaded group, whereas c-JUN was down-regulated in the nucleus and up-regulated in the annulus in the LDL group. IL6 was only up-regulated in the nucleus region. In the SPL group there was no significant change in expression of inflammation-related genes when compared to day 0 (figure 6).

### Extracellular matrix content

Mean water content (±SD) of fresh IVDs was 75.2% (±3.4%) in the nucleus and 58.4% (±4.0%) in the outer annulus. Mean water content in the IVD regions did not change significantly in any of the experimental groups at any time-point when compared to day 0 (table 2a).

**Table 2:** Mean water-content (table 2a; percentage water in tissue ± SD), mean GAG-content (table 2b; µgr GAG/mg dry weight) and mean collagen-content (table 2c; µgr hyp/mg dry weight) for day 0, unloaded, LDL and SPL group in the nucleus and outer annulus region at 7, 14 and 21 days of culture.

Table 2a. Mean water-content					
		Nucleus		Outer Annulus	
Control	day 0	75.2	±3.4	58.4	±4.0
Unloaded	7 days	73.4	±4.2	52.2	±8.7
	14 days	71.8	±1.8	55.9	±9.0
	21 days	73.0	±2.2	54.7	±6.0
LDL	7 days	73.8	±2.2	56.0	±5.3
	14 days	73.7	±2.0	55.2	±2.8
	21 days	72.6	±4.7	54.9	±3.2
SPL	7 days	70.9	±2.3	55.6	±2.9
	14 days	74.8	±4.0	56.2	±6.9
	21 days	72.8	±2.8	55.8	±4.4

Table 2b. Mean GAG-content					
		Nucleus		Outer Annulus	
Control	day 0	370.5	±46.8	78.8	±14.5
Unloaded	7 days	322.8	±51.3	76.2	±15.6
	14 days	329.1	±54.3	70.4	±10.7
	21 days	372.8	±35.2	75.8	±7.5
LDL	7 days	336.8	±53.7	82.0	±34.5
	14 days	340.6	±47.8	82.6	±26.8
	21 days	376.1	±67.8	80.1	±6.0
SPL	7 days	371.8	±107.3	75.0	±11.5
	14 days	364.6	±82.4	68.5	±9.7
	21 days	383.8	±77.0	80.1	±20.6

Table 2c. Mean collagen-content					
		Nucleus		Outer Annulus	
Control	day 0	16.5	±7.7	38.7	±11.3
Unloaded	7 days	17.8	±6.5	40.3	±10.2
	14 days	21.3	±5.9	42.5	±13.3
	21 days	20.4	±8.1	38.8	±16.9
LDL	7 days	15.9	±8.7	37.6	±6.5
	14 days	20.5	±9.1	41.0	±8.2
	21 days	13.8	±9.1	43.2	±8.2
SPL	7 days	18.7	±4.3	39.3	±16.0
	14 days	14.1	±4.1	38.7	±6.0
	21 days	16.5	±4.7	39.4	±15.2

No significant difference found (*p*≤0.05) when comparing experimental groups with the day 0 group in a linear mixed model with Bonferroni post-hoc testing.

Mean glycosaminoglycan (GAG) content (±SD) of day 0 samples was 370.5 µgr GAG/mg dw (±46.8 µgr GAG/mg dw) in the nucleus and 78.8 µgr GAG/mg dw (±14.5 µgr GAG/mg dw) in the outer annulus. Inner annulus values were intermediate and showed large variance (data not shown). GAG content varied between goats, but did not depend on disc level. Therefore, when comparing between experimental groups, the goat was included as a random factor in the model. For all regions and time points, mean GAG-content did not significantly change from the mean at day 0 in each of the experimental groups (table 2b).

Mean collagen content (±SD) of all day 0 samples was 16.5 µgr Hyp/mg (±7.7 µgr Hyp/mg) in the nucleus and 38.7 µgr Hyp/mg (±11.3 µgr Hyp/mg dw) in the outer annulus. Since collagen content varied slightly between goats, goats were included in the mixed model as a random effect. No significant differences in mean collagen content were measured when compared to day 0 (table 2c).

The values found for the inner annulus region for all matrix parameters were in between those of the nucleus and outer annulus and showed large variance, therefore this data set is not shown.

## Discussion

In this study, we have shown the feasibility of culturing lumbar goat IVDs with endplates in a Loaded Disc Culture System. We found that lumbar IVDs from adult goats could be maintained up to three weeks when subjected to simulated-physiological loading, whereas unloaded or low dynamically loaded discs showed deterioration of cell viability, cell density and gene expression.

Ex vivo culture of large IVDs is challenging and many factors have been identified to be critical for maintenance of IVD properties. Although especially NP cells have been reported to be robust and able to withstand harsh environmental conditions [47], for preservation of cell phenotype and metabolism a narrow optimum range for glucose [48], pH, oxygen [49]–[51] and osmotic pressure [52]–[54] has been reported. These environmental conditions could all be adequately maintained during IVD culture in the described custom-designed LDCS.

In our culture model we chose to preserve the endplates of the IVDs. By conserving the anchorage of the annular collagen lamellae within the endplates, the axial load is delivered in a physiological manner [55], [55], thereby preserving the biomechanical properties of the disc [42], [56]. The findings in this study confirm that IVD stiffness and disc height can be maintained when preserving the endplates. Also, previous studies showed that retaining the cartilaginous endplate does not hamper nutrient diffusion to the disc [57], [58], while it does constrain the disc tissue, preventing it from free swelling [36], [37], [39], [59], [60]. In our system, unloaded IVDs did not significantly change in height over the entire culture period. Swelling of unloaded specimens was probably prevented due to the containment of the tissue between the endplates and the osmolarity of the medium. Importantly, SPL loaded specimens did not show any significant change in IVD stiffness over time.

As with the other culture conditions, the effects of dynamic loading on biological parameters such as cell viability, have been reported to be within an optimum range [29], [61]–[63]. Here we showed that cell viability could be maintained for 21 days in the group that received simulated-physiological loading. We quantified cell viability by counting fluorescently stained cells on transverse cryosections, rather than using dissected tissue samples and analyses by confocal microscopy. Advantages of this method are that cell viability as well as the total number of cells can be quantified in clearly defined regions of the disc, due to conservation of IVD morphology. Changes in cell viability were first seen in the nucleus. In the unloaded group cell viability already drops significantly within the first week of culture, without a significant change in cell density. Although cell viability in the unloaded group seems to stabilize with increased culture duration, the drop in cell density reveals that overall disc vitality is still diminishing. Continuous low loading could not prevent cell death either.

Alterations in gene expression in response to different loading conditions were also more evident in nucleus cells than in the annulus. Here too, the most pronounced changes could be observed in the unloaded culture group. Absence of mechanical loading led to reduced expression of all anabolic genes except collagen type 1. This might be interpreted as a first sign of degenerative changes at the level of transcriptional activity, but also and more particularly of dedifferentiation of the nucleus cell population towards a fibrocartilaginous lineage [64], [65]. Remodeling genes as well as inflammation-related genes are known to be up-regulated in an adverse response of cells to loading [29], [48], [54], [62], [66]. Both the unloaded and LDL group showed significant up-regulation of expression of several of these target genes, especially in the nucleus. In the IVDs in the SPL group only slight changes in expression of remodeling genes were detected relative to baseline and no inflammation-related genes were up-regulated in this group. In comparison to the unloaded and LDL group, SPL loading most optimally preserved the expression of the studied genes relative to baseline.

Measured water, GAG, and collagen content in caprine discs correspond well with values reported for adult human discs [67]. We chose to express the GAG and collagen content relative to dry tissue weight as opposed to wet weight or total DNA, as dry tissue weight is an absolute measure. Wet weight and total DNA can be sensitive to confounding factors, such as changes in sample handling (wet weight) or the total number of live cells (total DNA). We could not detect any quantitative changes in matrix content for GAG and collagen. However, the sensitivity of the colorimetric assays used, may be insensitive to minor changes in matrix content. Nevertheless, histological sections did also not reveal changes in matrix staining between day 0 and day 21 of cultured discs. Longer culture periods may be needed to measure significant matrix content loss. For a more detailed analysis sensitive methods to detect matrix breakdown or turnover (qualitative or quantitative) could be used in future studies.

An absence or deficit of loading on the IVD caused pathological changes in the disc as is evident from a decline in cellular vitality and changes in gene expression patterns, especially in the nucleus. These findings are the resultant of both direct and indirect influences of mechanical loading on the IVD. Cells which in the *in vivo* situation receive abundant mechanical stimuli from the various forces on the IVD, are deprived of these stimuli in the unloaded culture group. A lack of hydrostatic pressure in combination with slightly hyper-osmotic medium, cause a different stress equilibrium compared to the physiological situation [54]. Indirect effects may involve a decrease of fluid flow by a deficit of deformation of the IVD in the unloaded and LDL state. This could impair distribution of nutrients towards and waste products from the nucleus [58].

The results in this study imply that a significant amount of dynamic loading is required to preserve cellular properties of caprine intervertebral discs. One can never fully separated what portion of this outcome can be attributed to the direct and indirect effects of mechanical loading to the discs. Whether these findings also apply to other IVD culture methods or even the *in vivo* human spine, remains speculative. Future research may be directed towards quantifying the effects of higher levels of loading, to assess whether these are stimulatory or detrimental for the IVD.

In conclusion, application of a simulated-physiological mechanical load proved essential for maintenance of caprine IVDs in the LDCS over a 21 day culture period. We believe that physical and biological processes involving cell-matrix interaction can only be studied in the intact lumbar IVD. Moreover, mechanical loading is required in order to mimic the physiological conditions of the IVD. The unique capabilities of the LDCS allows us to answer many important questions regarding biological processes involved with disc degeneration. Together with our well established *in vivo* goat disc degeneration model, this proposed *ex vivo* model will serve as a valuable tool to downscale the use of life animals and make pre-clinical *in vivo* testing of future therapeutics aimed against DDD more efficient.

## Materials and Methods

### Specimens and Culture Conditions

Thirty-five lumbar spines from skeletally mature (3–5 year-old) Dutch milk goats were obtained from a local abattoir (no approval of ethical board required). Within 3 hours of slaughter, IVDs with adjacent cartilaginous endplates (L1–L6) were dissected under sterile conditions using an oscillating surgical saw. Maximal width, depth (midsagittal), and height of the IVDs with endplates (EP) were measured with a caliper. The cross-sectional IVD area was calculated assuming an elliptic shape:
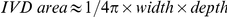



From each spine, two IVDs (Th13-L1 and L1–L2) were used as baseline reference for the parameters measured. The remaining IVDs were cultured over 7, 14, or 21 days in individual culture chambers of the LDCS, which is housed in an incubator at 37°C, 95% humidity, and 5% CO_2_. Discs were cultured in standard DMEM (Gibco, Paisley, UK) with 10% FBS (HyClone, Logan, UT), 4.5 gr/L glucose (Merck KGaA, Darmstadt, Germany), 50 µg/ml ascorbate-2-phosphate (Sigma Aldrich, St. Louis, MO), 25 mmol/L HEPES buffer (Invitrogen), 10,000 u/ml penicillin, 250 µg/L streptomycin, 50 µgr/mL gentamicin and 1.5 µgr/mL amphoterizin B (all from Gibco). Medium (40 ml per culture system) was circulated continuously (3 ml/hr) using a peristaltic pump and was exchanged every 48 hours and checked for pH (7.2–7.4) and osmolarity (360–380 mOsm; measured by cryoscopy).

### Loaded Disc Culture System

An overview picture of the LDCS actuators, a schematic picture of a single actuator and a detailed cross-section image of an a culture chamber are given in figure 1. The LDCS consists of two large incubators (Forma Steri-cult, Thermo Scientific, Asheville, NC), each housing twelve actuators which are individually controlled and monitored by a Labview-based custom built software program. Each actuator delivers force-controlled axial loading (clamping module EV63, Festo Corporation, Hauppage, NY) to a culture chamber, which is regulated via a feedback-loop system. IVD loading and displacement are continuously measured (Kam-e load cell, Bienfait, Haarlem, The Netherlands; oadm12 optoelectric sensor, Baumer, Berlin, Germany), signals are digitized (100 Hz) and stored in a PC for further analyses. The custom designed three piece culture chamber comprises of two similar top and bottom parts made of polycarbonate, with a central in- and output channel for the culture medium. A thin semi-transparent silicon membrane connects top and bottom halves. The IVD is placed in the center of the culture chamber with rigid titanium filters on each endplate. The center axial screw is set to make contact with the top part of the culture chamber, thereby adjusting for the individual height of the IVDs. This is done during real-time load measurement, making sure the screw is fitted without applying load to the disc (between 0–5 Newton maximum). Culture medium is pumped over the bottom endplate into the culture chamber, immersing the IVD. Medium exits over the top endplate into a high-surface, low-volume medium reservoir for optimal gas exchange with filter sterilized air.

### Loading Protocols and Mechanical Properties

Mechanical loading of the IVDs was strictly axial. Loading magnitudes (Newton; N) and frequency (Hertz; Hz) were derived from *in vivo* pressure measurements in a lumbar goat IVD during different activities, such as lying down, walking and jumping on a haystack (data not shown) [68]. IVDs were assigned to one of three experimental culture groups: 1. without loading (unloaded), 2. continuous low dynamic load (LDL; 0.1–0.2 MPa, 1 Hz) or 3. diurnal simulated physiological load (SPL) consisting of a sinusoidal load (1 Hz) alternating in magnitude every 30 minutes (0.1–0.2 MPa and 0.1–0.6 MPa) for 16 hours per day, followed by 8 hours of low dynamic load (0.1–0.2 MPa). The LDL and SPL loading conditions are approximations of the measured pressures during respectively lying down and walking. For standardization, the LDL and SPL regimes were preceded by a low dynamic load (sinusoidal; 0.1–0.2 MPa; 1 Hz) during the first 8 hours of culture, the SPL regime also ended with 8 hours of LDL loading.

The mean displacement at the end of each daily loading cycle was analyzed to assess overall disc height changes over time. In dynamically loaded discs, IVD stiffness was calculated from the load deformation curves of the ascendant part of 5 consecutive sine waves at consistent time intervals using regression.

### Histology and quantitative cell biology

Directly after dissection from the spine (baseline control) or culture in the LDCS, selected IVDs were fixed in 4% formaldehyde for 48 hours and decalcified for 10 days using standard Kristensen's fluid. Paramidsagittal tissue slices (3 mm thick) were cut from the IVD specimen with a scalpel and embedded in paraffin. With a microtome, 3 micrometer (µm) thin sections were cut and stained with safranin-O (proteoglycans) and Masson's trichrome (collagen).

Cell viability was assessed in the nucleus pulposus (NP), the inner (iAF) and outer annulus fibrosis (oAF). We removed one endplate and incubated IVDs (n≥6 for each group and time point) in a 6-well plate in serum-free medium containing 2 µM Celltracker Green (CTG; Chloromethylfluorescein, Molecular Probes, Eugene, OR) and 2 µM propidium iodide (PI; Sigma) under free-swelling conditions. After incubation for 1 hour, IVDs were washed in PBS, flash-frozen and 10 µm transverse cryosections were cut with a cryostat. Images (1048×1342 pixels) were taken at 10× magnification (surface area ∼1 mm^2^) using fluorescent light on an inverted microscope (Leica DM6000, Wetzlar, Germany; filters: I3 S450–490 nm and N2.1 S515–560 nm). The total number of cells per area (cell density) and the percentage of live cells (100% (# live cells/# total cells)) were determined using 10 images per region for each IVD. Co-labelled cells were counted for the cell density measurement, but were excluded from the analysis of cell viability. A fresh (day 0) IVD was used as positive control. As a negative control, a thoracic IVD, which underwent a freeze-thawing cycle three times prior to staining was used.

### RNA isolation, cDNA synthesis and RT-qPCR

Nucleus and outer annulus tissue samples were homogenized with ceramic beads in a lysis solution (MagnaLyser, GmbH, Roche Diagnostics, Brussels, Belgium) with 4 runs of 30 seconds at 6500 rpm with in-between cooling. Total RNA was isolated with the MagnaPure robot using the RNA isolation kit III (both Roche Diagnostics). cDNA synthesis was performed using Superscript Vilo® (Invitrogen, Merelbeke, België) and real-time PCR reactions were performed using the SYBRGreen reaction kit (Roch Diagnostics) both according to the manufacturer's instructions in a LightCycler 480 (Roche Diagnostics). IVD cell gene expression was assessed for a range of anabolic (collagen types 1, 2 and 6, aggrecan, biglycan, and Sox9), catabolic/remodeling (MMP (matrix metalloproteinase) 1, 13 and 14, ADAMTS (a disintegrin and metalloproteinase with thrombospondin motifs) 4 and 5, TIMP (tissue inhibitors of metalloproteinase) 1 and 3) and inflammatory-related genes (c-JUN, COX (cyclooxygenase) 2 and IL (interleukin) 6). The primers used for the gene expression analyses are shown in table 3. Stability of expression of housekeeping genes YWHAZ (tyrosine 3-monooxygenase/tryptophan 5-monooxygenase activation protein) and 18 S (ribosomal RNA) was calculated by geNorm software (http://medgen.ugent.be/genorm). As the expression of all genes was within a 3-fold range of YWHAZ expression levels, this housekeeping gene was used as normalization factor. Absolute expression of all genes was quantified with fitpoint calculation (Lightcycler software) using the standard curve method, based on serial dilution of standards for each gene. Relative gene expression is shown as the ratio between absolute expression of the gene of interest divided by the absolute YWHAZ expression of the same sample. Samples with no detectable RNA concentration of the target gene, but with detectable gene concentration of the housekeeping genes (Ct<18) were assigned a Ct of 45 (i.e. detection threshold).

**Table 3:** Primer sequences used for PCR.

Target gene		Oligonucleotide sequence	Annealing temperature (°C)	Product size (bp)
18 S	Forward	5′ GTAACCCGTTGAACCCCATT 3′	57	151
	Reverse	5′ CCATCCAATCGGTAGTAGCG 3′		
YWHAZ	Forward	5′ GATGAAGCCATTGCTGAACTTG 3′	56	229
	Reverse	5′ CTATTTGTGGGACAGCATGGA 3′		
Collagen 1a1	Forward	5′ TCCAACGAGATCGAGATCC 3′	57	191
	Reverse	5′ AAGCCGAATTCCTGGTCT 3′		
Collagen 2a1	Forward	5′ TGTCAGGGCCAGGATGT 3′	56	256
	Reverse	5′ CTCCTTTCTGTCCCTTTGG 3′		
Collagen 6	Forward	5′ CAGTGACGAGGTGGAGATCAT 3′	57	294
	Reverse	5′ ATGGCCACCGAGAAGAC 3′		
Aggrecan	Forward	5′ CAACTACCCGGCCATCC 3′	57	160
	Reverse	5′ GATGGCTCTGTAATGGAACAC 3′		
Biglycan	Forward	5′ TACAGCGCCATGTGTCCTT 3′	59	274
	Reverse	5′ GGTGGTTCTTGGAGATGTAGAG 3′		
Sox9	Forward	5′ CCCAACGCCATCTTCAAGG 3′	57	242
	Reverse	5′ CTGCTCAGCTCGCCGATGT 3′		
MMP1	Forward	5′ GTGGACCATGCCATTGAGAA 3′	56	387
	Reverse	5′ GGCTTGGATGCCATCAATGT 3′		
MMP13	Forward	5′ GGAGCATGGCGACTTCTAC 3′	56	208
	Reverse	5′ GAGTGCTCCAGGGTCCTT 3′		
MMP14	Forward	5′ CTGAGATCAAGGCCAATGTTC 3′	56	206
	Reverse	5′ CTCACGGATGTAGGCATAGG 3′		
ADAMTS4	Forward	5′ CATCCTACGCCGGAAGAGTC 3′	57	278
	Reverse	5′ GGATCACTAGCCGAGTCACCA 3′		
ADAMTS5	Forward	5′ GTGGAGGAGGAGTGCAGTTTG 3′	57	320
	Reverse	5′ TTCAGTGCCATCGGTCACCTT 3′		
TIMP1	Forward	5′ CACAGACGGCCTTCTGCAA 3′	57	211
	Reverse	5′ TTGTGGGACCTGTGGAAGT 3′		
TIMP3	Forward	5′ AGGACGCCTTCTGCAACTC 3′	57	163
	Reverse	5′ GCTTCCGTATGGATGTACTG 3′		
c-JUN	Forward	5′ GGATCAAGGCGGAGAGGAA 3′	57	232
	Reverse	5′ TGCAACTGCTGCGTTAGCAT 3′		
COX2	Forward	5′ AGACCAGGCACCAGACCAAAGA 3′	56	299
	Reverse	5′ GCATTCTTTGCCCAGCACTT 3′		
IL6	Forward	5′ CTCTTCACAAGCGCCTTCAGT 3′	57	248
	Reverse	5′ GCCAGTGTCTCCTTGCTGTT 3′		

Table 3. Primers used for the gene expression analyses showing the oligonucleotide sequences, annealing temperature and product size.

### Quantitative Biochemistry

Tissue samples (n≥12 for each group and time point) were taken from nucleus, inner annulus (iAF) and outer annulus (oAF) regions of the IVDs. Water content of each sample was calculated from measured wet (ww) and dry weights (dw), before and after freeze drying (speedvac). Dry weight samples (∼1 mg dw/sample) were digested in a papain-digestion buffer as previously described in Hoogendoorn *et al.* [69]. Papain-digestion suspension (10 uL) was analyzed using a DMMB assay (Biocolor Ltd., Carrickfergus, UK) in accordance with the manufacturer's description. Measured amount of GAG for each sample was normalized to tissue dry weight. From the remaining papain-digestion solution, 500 uL was used to quantify total collagen content using a DMBA hydroxyproline assay adapted from Hoogendoorn *et al.* [69]. A hydroxyproline calibration curve made with a standard solution (60 µg/ml hydroxyproline) was used to quantify sample content. Total collagen is expressed as micrograms hydroxyproline per milligram tissue dry weight.

### Statistical Analysis

All data was analyzed using linear mixed models. Separate analyses were performed for the three disc regions. Experimental outcome parameters were included as dependent variables in the models. The models included a fixed effect for test duration (for cell viability and cell density, water, GAG en collagen) or experimental loading condition (for gene expression). A random effect for each goat (for gene expression, water, GAG and collagen) or goat and IVD combination (for cell viability and cell density) was included in the model. The random effect was needed to account for correlation of measurements as a series of repeated measures were made for each goat. The same day 0 measurements (all that were available for the region considered) were included in the statistical analyses for each of the loadings. Bonferroni posthoc testing was used to compare mean outcome measures for each loading and test durations with baseline and groups and time points amongst each other.
